# Identification of functional modules that correlate with phenotypic difference: the influence of network topology

**DOI:** 10.1186/gb-2010-11-2-r23

**Published:** 2010-02-26

**Authors:** Jui-Hung Hung, Troy W Whitfield, Tun-Hsiang Yang, Zhenjun Hu, Zhiping Weng, Charles DeLisi

**Affiliations:** 1 Bioinformatics Program, Boston University, 24 Cummington Street, Boston, MA 02215, USA; 2 Department of Biochemistry and Molecular Pharmacology and Program in Bioinformatics and Integrative Biology, University of Massachusetts Medical School, 364 Plantation Street, Worcester, MA 01605, USA; 3 Department of Biomedical Engineering, 44 Cummington Street, Boston University, Boston, MA 02215, USA

## Abstract

One of the important challenges to post-genomic biology is relating observed phenotypic alterations to the underlying collective alterations in genes. Current inferential methods, however, invariably omit large bodies of information on the relationships between genes. We present a method that takes account of such information - expressed in terms of the topology of a correlation network - and we apply the method in the context of current procedures for gene set enrichment analysis.

## Abstract 2

A gene set enrichment analysis method for including network topology in the identification of genes involved in phenotypic alterations is described. Classifications: Genome studies, Methods

## Background

A central problem in cell biology is to infer functional molecular modules underlying cellular alterations from high throughput data such as differential gene, protein or metabolite concentrations. A number of computational techniques have been developed that use expression for class distinction to identify, from among *a priori *defined sets of functionally or structurally related genes, those that correlate with phenotypic difference (see, for example, Goeman and Buhlmann [1]). More sophisticated approaches have used random forests to capture nonlinear and complex information in expression profiles [2]; applied linear transformations to measure the discriminative information of genes [3]; and combined information from multiple assessments [4].

One of the most widely used methods, gene set enrichment analysis (GSEA) [5], ranks genes according to their differential expression and then uses a modified Kolmogorov-Smirnov statistic (weighted K-S test) as a basis for determining whether genes from a prespecified set (for example, Kyoto Encyclopaedia of Genes and Genomes (KEGG) pathways or Gene Ontology (GO) terms) are overrepresented toward the top or bottom of the list, correcting for false discovery when multiple sets are tested [6]. The central message of this paper is that discovery depends strongly on the type of correlation used, and we illustrate this point by elaborating on the biological implications of two different cancer data sets. GSEA uses a weighted Kolmogorov-Smirnov statistic (WKS) to quantify enrichment. The weight is related to the correlation with phenotype, essentially omitting known network properties of gene sets. Here we take such properties into account, as explained below. We reserve the term WKS for describing GSEA, and refer to our method, which integrates topological information, as pathway enrichment analysis (PWEA), where a pathway is defined as a pair of nodes connected by an uninterrupted set of intervening nodes and edges, such as those found in protein-protein interaction networks, signal transduction networks, and metabolic pathways. In this paper we use KEGG pathways. Just as WKS represents a conceptual and practical improvement over the K-S test, we show in this paper that the inclusion of topological weighting is not only a conceptual change in enrichment analysis, but a substantial practical improvement.

Several recently introduced techniques, including ScorePAGE [7], gene network enrichment analysis [8] and Pathway-Express [9], incorporate concepts of gene topology. ScorePAGE uses a topology-weighted cross-correlation of time-dependent (or condition-dependent) gene expression data to assign a significance value to *a priori *defined KEGG metabolic pathways. Gene network enrichment analysis first identifies a high-scoring transcriptionally affected sub-network from a global network of protein-protein interactions, and then identifies gene sets that are enriched in the sub-network using a Fisher test. Pathway-Express contains in its scoring function a term that increases the scores of the genes that are directly connected to other differentially expressed genes, which in turn produces a higher overall score for predefined KEGG signaling pathways in which the differentially expressed genes are localized in a connected sub-graph. Other strategies that extract enriched functional submodules [10,11] or paths [12] from protein-protein interaction networks or other topological pathways without strict boundary (that is, identify only a subset of networks without *a priori *gene set definition) also take advantage of the topology.

Here we present a new and general method for incorporating disparate data into statistical methods used to infer functional modules from a class distinction metric. In order to fix ideas and compare with the most popular method, we use differential expression to distinguish phenotype and define a *topological influence factor *(*TIF*) to weight the K-S statistic. The *TIF*, however, can just as easily be used with other kinds of class distinctions as data become available, and with other kinds of statistics.

The contributions of this paper are both methodological and biological. The methodological contribution consists of including known correlations among the genes in a gene set in the weighting procedure. When applied to cancer data sets we find that the inclusion of longer-range correlations substantially improves sensitivity, with little or no loss of specificity. In particular for colorectal cancer, PWEA and GSEA agree on 24 out of 25 pathways identified by GSEA, but PWEA identifies an additional 10 pathways, 8 of which, including oxidative metabolism of arachidonic acid, are supported by evidence from the literature. For small cell lung carcinoma, PWEA finds all 19 of the pathways identified by GSEA, and an additional 14 highly plausible pathways, including apoptosis, MAPK signaling pathway, Jak-STAT signaling pathway, and the GnRH signaling pathway.

## Results

### The topological influence factor

The goal of enrichment analysis is to discover sets of related genes that correlate with differential behavior. However, many such sets, including pathways and chromosomal locations in linkage disequilibrium, have long range correlations whose omission could affect conclusions. Thus, in an established biochemical pathway, nearest neighbor interactions are implicitly present in standard analysis, but cross-talk between pathways is missing, as is possible variation in correlation between non-neighboring genes that might be identified by genetic interactions, phylogenetic analysis and so on. Here, we define the correlation between genes in a network by an influence factor, Ψ. We constrain the functional form of Ψ by assuming that the influence of genes *i *and *j *on one another will drop as the ratio of the shortest distance between them to their correlation, the latter being obtained from variations in expression over a set of conditions. In particular, we define the mutual influence between two genes as:(1)

where *f*_*ij *_= *d*_*ij*_/|*c*_*ij*_|, *d*_*ij *_is the shortest distance between genes *i *and *j*, and *c*_*ij *_is the correlation based on their expression profiles. If *m *is the total number of samples, including both normal and disease samples, then the Pearson correlation coefficient is:

where *i*_*k *_is the expression level of gene *i *in sample *j*, and *s*_*i *_is the sample standard deviation of gene *i*. The exponential form of Equation 1 is suggested by the observed discriminative weight of each gene measured by the machine learning algorithm introduced in Fujita *et al*. [3]. It is reasonable to expect that only close neighbors with strong correlations will contribute significantly to the score.

Since *d*_*ij *_and |*c*_*ij*_| are positive definite, and positive, respectively, 0 < Ψ_*ij *_≤ 1, and Ψ behaves in an obvious and intuitive manner as shown in Figure S1 in Additional file 1. We further define the *TIF *of a gene *i *as the average mutual influence that the gene imposes on the rest of the genes in the pathway. In particular (see Materials and methods):(2)

where *n *is the total number of genes connected by paths starting at gene *i*. If *TIF*_i _is small, gene *i *fails to affect the pathway and its abnormality can be eliminated by genetic buffering (Additional file 1) or some other effect (see Discussion and conclusions). Otherwise, the gene could play an important role in perturbing the functionality of the pathway. Although we apply *TIF *only to KEGG pathways in this paper, its definition allows application to a general network.

### Controlling the magnitude of *TIF*

One shortcoming of Equation 2 is that the effect of a gene on a few nearby and tightly correlated genes can be washed out if the gene influences many other genes weakly (see Discussion and conclusions). In order to avoid this difficulty, we define a filtering process (see Materials and methods) to include only genes for which Ψ is larger than a given threshold, *α*. From observing the behavior of Ψ (Figure S2 in Additional file 1), *α *is set to 0.05. The final *TIF *is written as:(3)

where Θ is the step function (see Materials and methods) and  is the total number of genes connected by paths starting at gene *i *and for which Ψ is larger than α. We use *TIF *as a weight rather than a statistic, that is, we use the *TIF *scores of all genes.

There is no restriction on the type of statistic that *TIF *can modify, although in this work we restrict our analysis to a modification of WKS (that is, GSEA), as described in Materials and methods. Please note that the value of *TIF *in the following context is in the form of 1 + *TIF*, to accommodate to the usage of the weighting scheme in WKS (see Materials and methods). The general comparison with three other gene set level statistical tests (that is, mean, medium and Wilcoxon rank sum test as described by Ackermann and Strimmer [13]), are shown in Table S4 in Additional file 1. In most cases, *TIF *weighting led to higher sensitivity.

### Test with synthetic random input

Rigorous performance evaluation of enrichment methods is difficult in the absence of a gold standard [6,9,14]. At a minimum, however, we require that the likelihood of inferring perturbed pathways from randomly generated data be insignificant, and that the performance of our method be comparable to that of other methods. In our test, PWEA does not show biased *P*-values in a sample generated by 500 random phenotype shuffles of the small cell lung cancer dataset. The comparison with WKS and K-S tests is shown in Figure S3 in Additional file 1. PWEA yields a uniform distribution of *P*-values in a randomly generated null background, just as do other proven approaches. In addition, as explained below, our analyses of six test sets suggests that PWEA has substantial sensitivity advantages with no loss of specificity compared with GSEA (Additional file 2).

### Application to cancer datasets

Expression profiles for two human cancer/normal datasets - colorectal cancer and small cell lung cancer - were extracted from NCBI Gene Expression Omnibus (GEO) [15]. Of the 14 cancer types represented among the KEGG pathways, these two are among those whose currently available cancer expression data in the GEO database have adequate sample size for statistical testing.

### Case study I: colon cancer dataset

The dataset [GEO:GDS2609] [16] consists of 10 normal and 12 early onset colorectal cancer samples. Since the mutual influence (Equation 1) of two genes depends on the correlation between their expression levels, the *TIF *of a particular gene pair will differ from one data set to the next, even though their topological relationship in a pathway is invariant. For each data set, a *TIF *score is assigned to all genes in every pathway. For the colon cancer pathway dataset, the *TIF *averaged over all genes in all 201 KEGG pathways is 1.06 ± 0.008.

In the remainder of this paper, we illustrate how the use of *TIF*s can uncover relationships that would otherwise be missed. As a general observation we note that although the ten genes with highest *TIF*s over all KEGG pathways (Table 1) do not always rank high in terms of differential expression, their functional annotations in GO and KEGG -- carcinoma, calcium signaling, cell adherent, cytokine receptor, metabolic system -- are nevertheless consistent with a role in cancer.

**Table 1:** Ten highest *TIF *genes in the colorectal cancer dataset

Gene	*TIF*	*t*-score (*P*-value)	KEGG annotation	GO annotation (evidence code^a^)
*SLC25A5*	1.34	4.79 (2e-6)	Calcium signaling pathway Parkinson's disease Huntington's disease	**Function:** Adenine transmembrane transporter activity (TAS) **Process:** Transport (TAS)
*CCR7*	1.33	1.90 (0.06)	Cytokine-cytokine receptor interaction	**Function:** G-protein coupled receptor activity (TAS) **Process:** Chemotaxis (TAS) Elevation of cytosolic calcium ion concentration (TAS) Inflammatory response (TAS)
*VDAC1*	1.32	5.82 (6e-9)	Calcium signaling pathway Parkinson's disease Huntington's disease	**Function:** Protein binding (IPI) Voltage-gated anion channel activity (TAS) **Process:** Anion transport (TAS)
*TCF7L1*	1.32	6.02 (2e-9)	Wnt signaling pathway Adherens junction Melanogenesis Pathways in cancer Colorectal cancer Endometrial cancer Prostate cancer Thyroid cancer Basal cell carcinoma Acute myeloid leukemia	**Function:** Transcription factor activity (NAS) **Process:** Establishment or maintenance of chromatin architecture (NAS) Regulation of Wnt receptor signaling pathway (NAS)
*NCAM1*	1.32	5.80 (7e-9)	Cell adhesion molecules (CAMs)	**Process:** Cell adhesion (NAS)
*SERPING1*	1.32	7.60 (3e-14)	Complement and coagulation cascades	**Process:** Blood circulation (TAS)
*C1R*	1.32	4.70 (3e-6)	Complement and coagulation cascades Systemic lupus erythematosus	**Function:** Serine-type endopeptidase activity (TAS)
*PPID*	1.32	4.04 (5e-5)	Calcium signaling pathway Parkinson's disease Huntington's disease	**Function:** Cyclosporin A binding (TAS) Protein binding (IPI)
*HADH*	1.32	5.94 (3e-09)	Fatty acid elongation in mitochondria Fatty acid metabolism Valine, leucine and isoleucine degradation Geraniol degradation Lysine degradation Tryptophan metabolism Butanoate metabolism Caprolactam degradation	**Function:** 3-hydroxyacyl-CoA dehydrogenase activity (EXP, TAS)
*GOT1*	1.30	3.69 (0.0002)	Glutamate metabolism Alanine and aspartate metabolism Cysteine metabolism Arginine and proline metabolism Tyrosine metabolism Phenylalanine metabolism Phenylalanine, tyrosine and tryptophan biosynthesis Alkaloid biosynthesis I	**Function:** L-aspartate:2-oxoglutarate aminotransferase activity (EXP, IDA) **Process:** Aspartate catabolic process (IDA) cellular response to insulin stimulus (IEP) response to glucocorticoid stimulus (IEP)

^a^Evidence codes defined by GO: EXP (Inferred from Experiment), IDA (Inferred from Direct Assay), IEP (Inferred from Expression Pattern), IPI (Inferred from Physical Interaction), NAS (Non-traceable Author Statement), and TAS (Traceable Author Statement).

A more specific observation is the high *TIF *but low *t*-score for the chemokine receptor CCR7 (Table 1). Its ligands, CCL19 and CCL21, also have high *TIF *scores (1.20 and 1.19, respectively). This finding is reinforced by the biological relationship among the three in immune reactions and lung disorders [17]. Indeed, both receptor-ligand complexes are implicated in colon cancer, cell invasion and migration [18].

More generally, by weighting genes according to their differential expression and longer range correlations, sensitivity for discovering perturbed pathways in colon cancer increases. In particular, we identified 34 pathways using a false discovery rate (FDR) below 0.01 (see Materials and methods). We applied GSEA to the same dataset and discovered 25 pathways, 24 of which were among the 34 identified by PWEA (Table S1 in Additional file 1).

The only pathway identified by GSEA and not by PWEA is the Adipocytokine signaling pathway. Polymorphism of adipokine genes such as *LEPR *can increase the risk of colorectal cancer [19]. Although *LEPR*'s relatively high *TIF *(1.15) indicates that it does perturb the network, the pathway does not have a high overall significance. PWEA may fail to discover this pathway due to its incompleteness, lacking either edges or nodes, which leads to many false 'extrinsic' genetic buffering effects (see Discussion and conclusions). Ten additional pathways found exclusively by PWEA are listed in Table 2, with independent evidence. Below, we discuss two examples that are especially striking.

**Table 2:** Pathways from the colon cancer dataset found exclusively by PWEA

Pathway	Size	DE fraction^a^	Type	Possible relation to the cancer	Reference.
Arachidonic acid metabolism	50	34%	Lipid metabolism	Inflammation Cell growth, related to MAPK signaling pathway	[20-22,72]
Axon guidance	126	20%	Development	Cell mobility and cell growth, related to MAPK signaling pathway	[28,32]
Nicotinate and nicotinamide metabolism	23	22%	Metabolism of cofactors and vitamins	Stimulate cell growth	[73,74]
Drug metabolism - cytochrome P450	63	30%	Xenobiotics biodegradation and metabolism	Therapeutic target, related to prognosis	[75]
Urea cycle and metabolism of amino groups	28	39%	Amino acid metabolic	Nutrition intake	[76]
Pyruvate metabolism	41	37%	Carbohydrate metabolism	Nutrition intake	[76]
Bile acid biosynthesis	31	39%	Lipid metabolism	Lead to high concentration of bile acid Resistance to bile-acid induced apoptosis	[77,78]
Colorectal cancer	84	15%	Disease	-	-
Long-term depression	70	15%	Disease	Unknown	-
Amyotrophic lateral sclerosis	54	15%	Disease	Inflammation and MAPK signaling pathway	-

^a^DE fraction is the fraction of genes that show differential expression with *P *< 0.05 using a two-tailed *t*-test.

#### Arachidonic acid oxidative metabolism pathway

Briefly, arachidonic acids (AAs) are essential fatty acids that are released from membrane phospholipids by phospholipase A_2 _in response to chemical or mechanical signals at the cell surface. The hydrolyzed AAs initiate a cascade of three signaling pathways that produce eicosanoids, a family of lipid regulatory molecules that includes prostaglandins and thromboxanes (when AA is a substrate for cyclooxygenase (COX)), various oxygenated states of the leukotrienes (when AA is a substrate for lipoxidase), and three types of P450 epoxygenase-derived eicosanoids.

**Figure 1:**
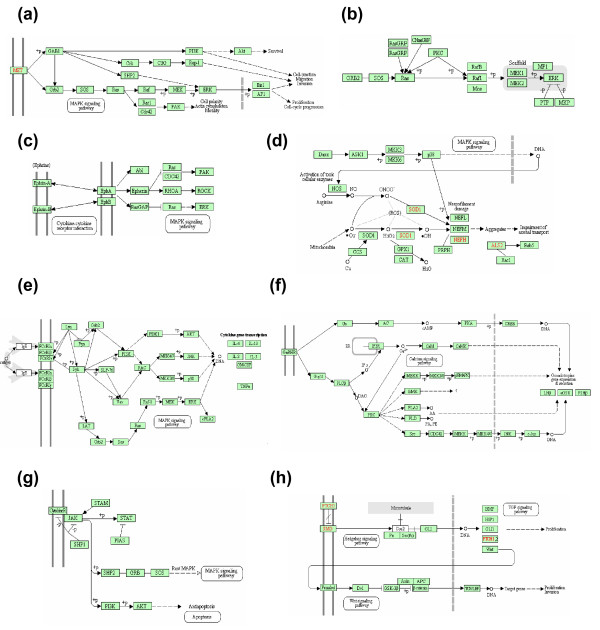
**Pathways adapted from KEGG**. **(a) **Renal cell carcinoma. **(b) **MAPK signaling pathway. **(c) **Axon guidance. **(d) **Amyotrophic lateral sclerosis. **(e) **Fcε RI signaling pathway. **(f) **Gonadotropin-releasing hormone signaling pathway. **(g) **Jak-STAT signaling pathway. **(h) **Basal cell carcinoma. Red indicates an abnormality.

Each of these pathways - the COX sub-pathway, the lipoxidase pathway and the epoxygenase pathway - have been implicated in several human cancers, including colon cancer [20]. The latter pathway is especially interesting because various P450 cytochromes are essential to it. In particular, CYP2J2 metabolizes epoxygenase-derived eicanosoids from AA into four *cis*-epoxyeicosatrienoic acids (EETs), 5,6-EET, 8,9-EET, 11,12-EET, and 14-15 EET [21]. These molecules have been shown to be involved in cancer pathogenesis by affecting various physiological processes, including intracellular signal transduction, proliferation (likely through the Erk/mitogen-activated protein kinase (MAPK) signaling pathway [20]; Figure 1b), inflammation [22], and inhibition of apoptosis. *CYP2J2 *has the highest *TIF *score (1.17) in this pathway. Other evidence suggests that *CYP2J2 *and EETs, which lead to phosphorylation of the epidermal growth factor receptor and the subsequent activation of downstream phosphoinositide 3-kinase (PI3K)/AKT and MAPK signaling pathways, suppresses apoptosis and up-regulates proliferation in carcinoma [23].

Genes in the COX pathway also show high *TIF *scores, such as *PTGS1 *(that is, *COX1*), *PTGS1 *(*COX2*), and *PTGIS *(1.12, 1.15, and 1.12, respectively). Similarly, genes with high *TIF *scores can also be observed in the lipoxidase sub-pathway, especially the arachidonate lipoxygenase family (*ALOX*), most of whose members have *TIF *scores above 1.09. The large number of genes showing high *TIF *scores indicates a significant tumor-associated perturbation.

#### Axon guidance pathway

There are four categories of axon guidance molecules (netrins, semaphorine, ephrine and members of the *SLIT *family) and their specific signal transduction routes comprise the axon guidance pathway. Briefly, netrin-1 (*NTN1*), the DCC family of receptors and the human *UNC5 *ortholog comprise part of a signaling pathway that is involved in the regulation of apoptosis, and whose dysregulation has been implicated in human cancers [24,25]. The *SLIT *family is involved in cell migration, so one might expect that aberrant or aberrantly expressed genes could contribute to metastasis, and that they will in any case affect migration of immune cells, which could predispose toward, or exacerbate, various disorders. In fact, the pathway involving *SLIT *and its roundabout receptor (ROBO) has been implicated in cervical cancer [26]. *SLIT2 *appears to be a candidate for a colon cancer suppressed gene, since it is often inactivated by LoH and hypermethylation [27] and its receptor, ROBO1, has been implicated in colon cancer [28], although the underlying mechanism of the *SLIT-ROBO *involved tumor growth remains obscure.

The *SLIT1*, *SLIT2 *and *ROBO1 *genes have significantly high *TIF*s: 1.18, 1.16 and 1.16, respectively. We also found that other receptors in axon guidance, such as *PLXNA1*, have high *TIF *scores (1.21). Our observations indicate a strong connection between colon cancer and axon guidance. Indeed, it has become evident that the axon guidance pathway reveals the critical roles that axon guidance molecules play in the regulation of angiogenesis, cell survival, apoptosis, cell positioning and migration [29-31]. It has been suggested that axon guidance shares a common mechanism with tumorigenesis, such as p53-dependent apoptosis [24,25].

Finally, the EphA family of axon guidance genes is known to be associated with the Ras/MAPK signaling pathway to control cell growth and mobility [32]; this pathway is also included in KEGG's axon guidance pathway. By examining the genes in the path leading from EphA to the MAPK signaling pathway (Figure 1c), we found that the MAPK signaling-related genes *EphA*, *RasGAP*, *Ras*, and *ERK *all have significant *TIF *scores (1.13, 1.15, 1.10, and 1.20, respectively). This finding implies that another candidate modulator of the abnormal behavior of colon cancer cell growth and cell mobility is linked to the MAPK signaling pathway.

**Figure 2:**
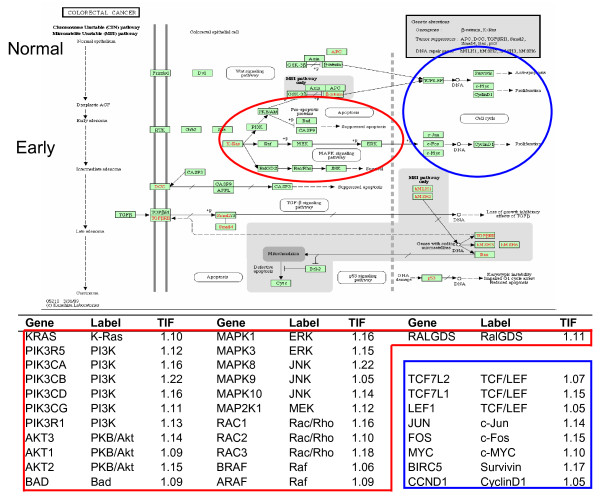
***TIF *scores for genes in the KEGG colorectal cancer pathway**. The regions circled in red and blue are clustered around the early stages of carcinoma, in accordance with the tissue origin being early stage.

We used KEGG to visualize the flow of physiological alterations associated with early stage adenoma. As indicated in Figure 2, most of the high *TIF *genes in the associated table are clustered in the upstream region of the MAPK signaling pathway in an apoptosis cluster (circled in red), and in a set of cell cycle genes (circled in blue). No gene with a high *TIF *score occurs in the late stage of the disease. This observation follows the expected behavior of genes from the samples, since they were collected from colonic mucosa at an early stage (Dukes A/B) [16]. These physiologically important clusters would not be identifiable by gene expression without the information provided by *TIF*.

The non-obvious associations of long-term depression and amyotrophic lateral sclerosis (ALS) with colorectal cancer are consistent with the idea that a particular aberrant gene or gene set can be implicated in distinctly different phenotypes [33]. Thus, superoxide dismutase (*SOD1*;*TIF *= 1.13, *t*-score = 5.04), which converts harmful superoxide radicals to hydrogen peroxide and oxygen, helps prevent DNA damage and is a possible cancer therapeutic target [34], and also impinges on the ALS pathway (Figure 1d). Genes related to MAPK signaling, particularly *p38 kinase*, which regulates neurofilament damage, have elevated *TIF *scores. It may be that the underlying mechanisms of ALS and early stage colorectal carcinoma are similar.

The results also suggest an association between colon cancer and renal cell carcinoma. PWEA and GSEA both report significant *P*-values for the KEGG renal cell carcinoma pathway; however, PWEA provides additional and more specific information. Genes with high *TIF *scores tend to cluster around the paths shown in Figure 1a. One of the paths influencing proliferation starts at the well-known oncogene *MET *(which encodes a Met tyrosine kinase and is present in both colorectal and renal cancer), and includes a sequence of genes that all have significant *TIF *scores: *GAB1*, *SHP2*, *ERK*, *AP1 *(*TIF *= 1.14, 1.23, 1.15, and 1.16, respectively). Similarly, another path from *MET *(dashed lines in Figure 1a) that influences survival, migration, and invasion includes *GAB1*, *PIK3*, and *AKT*, each of which has a significantly high *TIF *score (1.14, 1.25, and 1.17, respectively). The high *TIF *scores of these genes in these pathways, which are common to colon and renal cancer, indicate a previously unreported overlap in the genes underlying changes in proliferation, invasion, and migration for these two cancers.

### Case study II: small cell lung cancer dataset

The small cell lung cancer dataset consists of 19 normal and 15 primary small cell lung cancer samples collected from [GEO:GSE1037] [35]. The ten genes with highest *TIF *scores among 201 pathways are listed in Table 3. These genes are associated with cell cycle (growth and division), apoptosis, immune response and metabolic pathways. The average *TIF *score of all genes is 1.07 ± 0.008. For two of the ten genes, *SPCS1 *and *BTD*, both from the biotine metabolism pathway, we found no direct evidence for association with lung cancer, nor is the biotine metabolism pathway discovered by PWEA (FDR > 0.01). These high *TIF *scores could be the result of a small number of neighbors passing the filtering process, which would make the result unreliable (see Materials and methods). Such an apparently local, false signal is unlikely to lead to false positive pathways since a significant pathway requires consistent global evidence in order to be observed with WKS (see Materials and methods).

**Table 3:** Ten highest *TIF *genes in the small cell lung cancer dataset

Gene	*TIF*	*t*-score (*P*-value)	KEGG annotation	GO annotation (evidence code^a^)
*SPCS1*	1.33	3.87 (0.0001)	Lysine degradation Biotin metabolism	**Function:** Molecular_function (ND) **Process**: Proteolysis (TAS)
*BTD*	1.33	5.60 (2e-8)	Biotin metabolism	**Function:** Biotin carboxylase activity (TAS) **Process**: Central nervous system development (TAS) Epidermis development (TAS)
*SKP2*	1.33	10.60 (3e-26)	Cell cycle Ubiquitin mediated proteolysis Pathways in cancer Small cell lung cancer	**Function:** Protein binding (IPI) **Process**: G1/S transition of mitotic cell cycle (TAS) Cell proliferation (TAS)
*CKS1B*	1.33	5.31 (1e-7)	Pathways in cancer Small cell lung cancer	**Process**: Cell adhesion (NAS)
*NFKB1*	1.29	5.69 (1e-8)	MAPK signaling pathway Apoptosis Toll-like receptor signaling pathway T cell receptor signaling pathway B cell receptor signaling pathway Adipocytokine signaling pathway Epithelial cell signaling in *Helicobacter pylori *infection Pathways in cancer Pancreatic cancer Prostate cancer Chronic myeloid leukemia Acute myeloid leukemia Small cell lung cancer	**Function**: Promoter binding (IDA) Protein binding (IPI) Transcription factor activity (TAS) **Process:** Anti-apoptosis (TAS) Apoptosis (IEA) Inflammatory response (TAS) Negative regulation of cellular protein metabolic process (IC) Negative regulation of cholesterol transport (IC) Negative regulation of IL-12 biosynthetic process (IEA) Negative regulation of specific transcription from RNA polymerase II promoter (IC) Negative regulation of transcription, DNA-dependent (IEA) Positive regulation of foam cell differentiation (IC) Positive regulation of lipid metabolic process (IC) Positive regulation of transcription (NAS)
*IL1R1*	1.29	11.07 (2e-28)	MAPK signaling pathway Cytokine-cytokine receptor interaction Apoptosis Hematopoietic cell lineage	**Function:** Interleukin-1, Type I, activating receptor activity (TAS) Platelet-derived growth factor receptor binding (IPI) Protein binding (IPI) Transmembrane receptor activity (TAS) **Process**: Cell surface receptor linked signal transduction (TAS)
*FCGR2B*	1.29	7.36 (2e-13)	B cell receptor signaling pathway Systemic lupus erythematosus	**Function:** Protein binding (IPI) **Process**: Immune response (TAS) Signal transduction (TAS)
*INPP5D*	1.29	12.69 (7e-37)	Phosphatidylinositol signaling system B cell receptor signaling pathway Fc epsilon RI signaling pathway Insulin signaling pathway	**Function:** Inositol-polyphosphate 5-phosphatase activity (TAS) Protein binding (IPI) **Process**: Phosphate metabolic process (TAS) Signal transduction (TAS)
*ST3GAL4*	1.29	5.07 (4e-7)	Glycosphingolipid biosynthesis - lacto and neolacto series	**Function:** Beta-galactoside alpha-2,3-sialyltransferase activity (TAS)
*BAAT*	1.29	0.52 (0.60)	Bile acid biosynthesis Taurine and hypotaurine metabolism Biosynthesis of unsaturated fatty acids	**Process**: Bile acid metabolic process (TAS) Digestion (TAS) Glycine metabolic process (TAS)

^a^Evidence codes defined by GO: ND (No biological Data available), EXP (Inferred from Experiment), IC (Inferred by Curator), IDA (Inferred from Direct Assay), IEA (Inferred from Electronic Annotation), IEP (Inferred from Expression Pattern), IPI (Inferred from Physical Interaction), NAS (Non-traceable Author Statement), and TAS (Traceable Author Statement).

PWEA reports 33 pathways; GSEA reports 19, all of which are among those found by PWEA (Table S1 in Additional file 1). As discussed by Subramanian and colleagues [6], the independent evidence that the 19 pathways are involved in small cell lung carcinomas is strong. The additional pathways uniquely discovered by PWEA are listed in Table 4 accompanied by evidence from the literature. From among the pathways listed in Table 4, we discuss three pathways that are especially intriguing.

**Table 4:** Pathways from the small cell lung cancer dataset found exclusively by PWEA

Pathway	Size	DE fraction^a^	Type	Possible relation to the cancer	Reference
GnRH signaling pathway	78	37%	Endocrine system	Negative autocrine regulator	[43,79]
Complement and coagulation cascades	56	54%	Immune system	Inflammation Metastatic and invasive properties	[80]
MAPK signaling pathway	199	38%	Signal transduction	Cell growth	-
Fc epsilon RI signaling pathway	63	44%	Immune system	Angiogenesis Inflammation	[37,41,42]
Apoptosis	67	34%	Cell growth and death	Apoptosis	-
ABC transporters	34	24%	Membrane transport	Drug resistance	[81]
Jak-STAT signaling pathway	93	37%	Signal transduction	Cell growth	[47-49]
Drug metabolism - cytochrome P450	41	51%	Xenobiotics biodegradation and metabolism	Anticancer drugs topotecan and etoposide	[75]
Drug metabolism - other enzymes	28	46%	Xenobiotics biodegradation and metabolism	Anticancer drug irinotecan	[75]
Histidine metabolism	24	42%	Amino acid metabolism	Nutrition intake. Small cell lung cancer marker, DDC involved.	[82,83]
Tryptophan metabolism	36	39%	Amino acid metabolism	As above	[82,83]
Phenylalanine metabolism	13	54%	Amino acid metabolism	As above	[82,83]
Fatty acid metabolism	37	38%	Lipid metabolism	Apoptosis. Therapeutic target	[84,85]
Basal cell carcinoma	36	17%	Disease	Proliferation invasion through hedgehog signaling pathway	-

^a^DE fraction is the fraction of genes that show differential expression with *P *< 0.05 using a two-tailed *t*-test. DDC: enzymatic neuroendocrine markers L-DOPA decarboxylase.

#### FcεRI signaling pathway

The FcεRI signaling pathway triggers signaling cascades of various effector and immunomodulatory functions related to inflammation in mast cells [36]. FcεRI responds to immunoglobulin E (IgE) activation and signals mast cells to work as effectors (by releasing histamine, proteases, and proteoglycans) and immunomodulators (by releasing proinflammatory and immunomodulatory cytokines, such as TNFα, IL1, IL2, IL3, IL4, IL6, and IL13 [37]. These cytokines recruit additional leukocytes - including T cells, B cells, macrophages and granulocytes - thereby promoting immune protection, whether against foreign or transformed self antigens [38]. Recent evidence suggests that cancer-related inflammation is among the key physiological changes associated with cancer, promoting proliferation, angiogenesis and metastasis [39].

The intrinsic inflammation pathway of tumor cells activated by genetic alterations releases chemokines and cytokines to create an inflammatory microenvironment, which stimulates leukocyte recruitment [40]. Although the Fcε RI signaling pathway in KEGG is constructed based on the immune responses of mast cells, it may be that this pathway is utilized by tumor cells to promote inflammation. Genes with high *TIF *values include the tyrosine kinases *Lyn*, *Syk*, *PI3K*, *PDK1*, and *AKT*, several of which tend to be specific to hematopoietic cells, and are components of signaling cascades leading from the plasma membrane to the nucleus, ultimately regulating the transcription of various cytokines, including TNFα (Figure 1e). Genes along another signaling route, including *Lyn*, *Syk*, *LAT*, *Grb2*, *Sos*, *Ras*, *Raf*, *MEK *and *ERK*, also show high *TIF *scores. Indeed, this *Ras-Raf *signaling path has been suggested to be the trigger for the production of inflammatory chemokines and cytokines in cancer cells [41,42], although our *TIF *scores also implicates the first route.

#### Gonadotropin-releasing hormone signaling pathway

Gonadotropin-releasing hormones (GnRHs) are development and growth related, and the GnRH signaling pathway has been implicated in several types of cancer [43]. Genes encoding proteins of the signal transduction path originating at the GnRH receptor and proceeding through LH, FSH, Gq/11, PLCβ, PKC, Src, CDC42, MEKK, MEK4/7, JNK, c-Jun, and other nodes in the JNK/MAPK signaling pathway (Figure 1f) all have relatively high *TIF *scores. The same is true of transduction through Gs, AC, PKA, and CREB toward LHβ and FSHβ, suggesting that both routes play a role in small cell carcinoma. Interestingly, although small cell lung cancer cells are known to secrete peptide hormones [44], mainly adrenocorticotropic hormone, there are only a few reports of ectopic production of gonadotropin by lung cancer cells [45,46]. The role of the GnRH pathway in controlling the production of gonadotropin in tumor cells remains poorly understood; our results suggest the possibility that small cell lung cancer cells hijack this pathway to help achieve autocrine modulation of their own proliferation.

#### Jak-STAT signaling pathway

The Jak-STAT signaling pathway is related to cell growth; it has been implicated in several kinds of cancers, so its identification is not surprising. This pathway is noted here primarily to contrast PWEA's sensitivity with that of the WKS test. Signaling proceeds from the plasma membrane through most of the genes with high *TIF *scores, prior to reaching the apoptosis pathway (Figure 1d), which is also found by PWEA (Table 4). Indeed, it has been shown that the STAT3-dependant growth arrest signal is inactivated in small cell lung cancer cells, resulting in growth promotion [47-49]. The fact that multiple perturbed pathways are related to cell growth is precisely what is expected for transformed cells.

Our results also show enrichment of differentially expressed genes in the basal cell carcinoma pathway, suggesting possible co-morbidity of basal cells and lung cancer. As this connection is not an intuitive one, we examined the genes with high *TIF *scores, and found that they were clustered in the Hedgehog and Wnt signaling pathways -- both developmental pathways that, when inappropriately activated, contribute to tumor progression. Several of the key inducers of the Hedgehog signaling pathway, *GLI1*, *GLI2 *and *GLI3*, have elevated *TIF *scores (1.12, 1.12, and 1.14, respectively). This pathway is important in proliferation and growth (Figure 1h) and *GLI1 *has been implicated in basal cell carcinoma in mice [50]; more generally, abnormal activity of *hedgehog-GLI *is associated with a variety of tumor types [51]. The coexistence of basal cell carcinoma and metastatic small cell lung cancer has been reported [52], although without a pathway level connection (Figure 1h).

**Figure 3:**
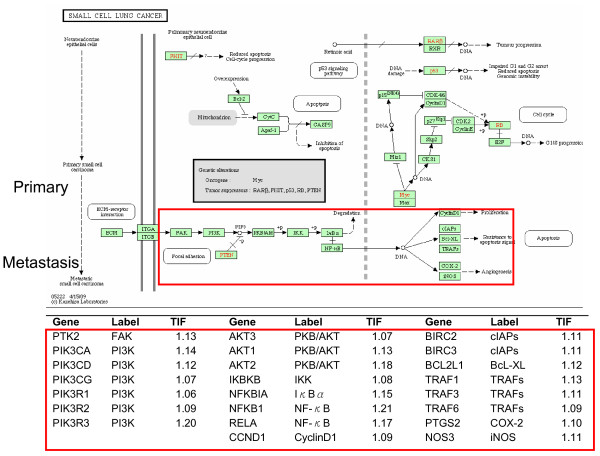
***TIF *scores for genes in the KEGG small cell lung cancer pathway**. The identification of genes associated with primary and metastatic stages is consistent with the tissue of origin being stage heterogeneous, and not purely primary.

Although the small cell lung cancer pathway can be identified by either PWEA or the WKS test, the distribution of high *TIF *genes provides additional information. While the samples were primary small cell lung cancer, the genes with high *TIF *scores cluster mainly between the primary and metastatic stages (Figure 3). Since lung cancer often metastasizes, the possible presence of tissue suggesting metastasis is not surprising, and illustrates the information content in *TIF *scores.

### Application to other datasets

In order to demonstrate the general utility of the method, we applied PWEA to four additional data sets that represent diverse biological processes: ovarian endometriosis [53], rheumatoid arthritis [54], Parkinson's disease [55], and sex [6]. The pathways discovered by PWEA on these additional data sets are listed in Tables S1 and S3 in Additional file 1. For the ovarian endometriosis dataset, PWEA reported all 33 pathways found by GSEA and 9 additional pathways. Published literature supports some of the newly identified pathways, including complement and coagulation cascades [56], purine metabolism [57] and sphingolipid metabolism [58]. For the rheumatoid arthritis dataset, GSEA found no pathways, while PWEA found the antigen processing and presentation pathway, reflecting the autoimmune nature of rheumatoid arthritis [59]. For the Parkinson's disease dataset, both PWEA and GSEA found only the vascular endothelial growth factor signaling pathway [60], which has been suggested to mediate mechanisms related to neuroprotection in rats with Parkinson's disease. In the sex dataset, PWEA and GSEA correctly report no pathways, indicating no significant difference between males and females. In general, PWEA discovered all pathways found by GSEA and uncovered additional biologically relevant pathways.

## Discussion and conclusions

Pathway enrichment analysis has been introduced as a method to interpret differential expression using not only *a priori *defined gene sets, but also the topological properties of the surrounding network. PWEA uses gene sets from the KEGG database to compute a *TIF *that describes the average mutual influence of neighboring genes within a pathway, including the effects of genetic buffering. Because the *TIF *is computed for one pathway at a time, PWEA cannot detect genetic buffering exerted by genes from outside a given pathway [61]; nor can any existing gene set analysis method. The calculation of *TIF *largely depends on the correlation of the expression levels of neighboring genes, which can be affected by small sample size. Moreover, if genes, or topological relationships between genes, are missing from the *a priori *defined gene sets used with PWEA, the method may fail to accurately assign statistical significance to some pathways. Any method attempting to interpret microarray data using *a priori *defined gene sets, however, faces a similar challenge.

Although genetic buffering relationships are not explicitly annotated in KEGG gene set topology, as they are in Figure S1b in Additional file 1, PWEA uses *TIF *to approximate their effects. Genes with low *TIF *values may have their influence in the network reduced by genetic buffering effects or by the incompleteness of the topology. *TIF *measures the effects of pathway topology on the biological function of individual genes. Genes receive a higher *TIF *if they are connected to other correlated differentially expressed genes nearby, regardless of the direction of those connections. PWEA does not, at present, take account of directionality. In principal, PWEA may be applied in a variety of contexts: given as input a score (*r*) for each gene with signature (phenotype), and the corresponding networks (pathways), PWEA can determine a significance value. Finally, by using the WKS framework, PWEA reduces to GSEA when topological information is absent, which means that PWEA is also applicable to GO enrichment analysis or any other predefined gene sets.

When applied to two cancer datasets, PWEA has shown a high specificity and ability to discover perturbed pathways. Examination of the pathways discovered by PWEA reveals that most are consistent with previously reported experimental findings. As would be expected of any method designed to aid in the interpretation of expression data, the pathways reported in PWEA give insights into the nature of the different types of cancer that were examined.

One of the potential problems with the method presented here is the requirement for accurate topology to calculate *TIF *scores. Pathways with missing genes or incomplete gene topology can lead to dramatically reduced *TIF *scores; gene set incompleteness can account for this behavior. Indeed, this feature of PWEA might be used in the future to aid in the refinement of existing pathway topologies.

It has become clear that pathways rather than individual genes are essential in understanding carcinoma [62,63]. PWEA has been shown to be effective at discovering biologically relevant pathways in cancers, making it a useful addition to the growing library of techniques for interpreting molecular profiling data.

## Materials and methods

PWEA requires three inputs: the expression profiles of two phenotypes, a list of gene sets, and their topology. In this study, the gene sets are taken from the KEGG database [64] as of April 2009: the gene files specify genes in a pathway and the map files encode topology, which in this case comprises the molecular interactions dictated by the pathway. In total, 201 KEGG pathways were included. Although we use KEGG pathways for convenient illustration, pathway data from other sources may also be annotated in the KEGG markup language (KGML) [65].

We denote the genes in pathway K by '*P*_*K*_', and all genes not in pathway K by 'Not *P*_*K*_'.

**Figure 4:**
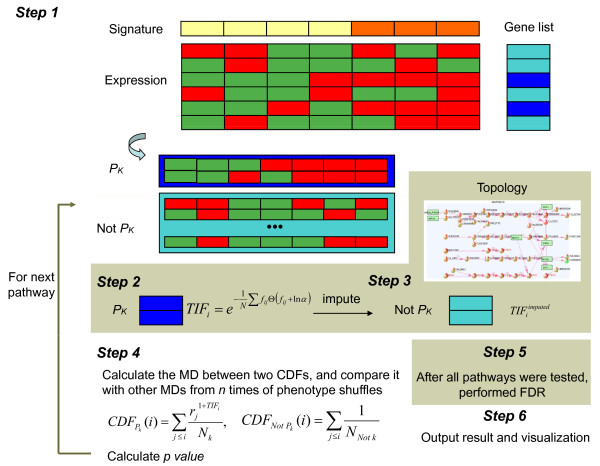
**Algorithmic scheme of PWEA**. In step 1, two different colors (yellow and orange) in the signature vector indicate two phenotypes (for example, normal and cancer). Blue rectangles in the gene list vector indicate genes in a particular pathway *P*_*k*_. For a pathway *k*, the expression profiles are categorized into two groups: *P*_*k *_(blue) and its complement, 'Not *P*_*k*_' (cyan). In step 2 the *TIF *scores for genes in *P*_*k *_are calculated. In step 3, *TIF *scores of the genes in 'Not *P*_*k*_' set is computed. In step 4, the maximum deviation (MD) between two cumulative distribution functions is computed. After calculating MD for each of *n *iterations of phenotype shuffling, the fraction of occurrences of shuffled MDs ≥ the original MD is the *P*-value of *P*_*k*_. In step 5, after all pathways have been tested, FDR is used to correct for multiple testing. In step 6, results and a KEGG markup language topology file for visualization in visANT [68] are the final output. CDF, cumulative distribution function.

The procedure consists of six steps (Figure 4).

### Step 1

Transform normalized expression levels into an expression matrix, and phenotypes into a signature vector, with genes corresponding to the rows and phenotypes corresponding to the columns of the expression matrix. Parse gene-set and map-files of KEGG pathways. Some nodes of KEGG pathways denote protein complexes or families. The corresponding genes are parsed separately and each is assigned the same connectivity and topological location as the parent node.

### Step 2

For a pathway K, compute a *TIF *score for each gene in *P*_*K*_. *TIF *is defined as the average of the mutual influence, Ψ, with all other reachable genes in the pathway. Ψ_*ij *_is used to evaluate the influence between the *i*th gene and the *j*th gene in *P*_*K*_, according to both the absolute value of the correlation of their expression patterns and their topological distances. Ψ_*ij *_is defined as:

where *f*_*ij *_= *d*_*ij*_/|*c*_*ij*_|, *d*_*ij *_is the shortest distance between gene *i *and gene *j *calculated using the Floyd-Warshall algorithm [66] (with *d*_*ii *_= 0), and *c*_*ij *_is the Pearson correlation coefficient between gene *i *and gene *j *based on their expression profiles over both normal and diseased tissues (also see the Results section). The *TIF *for a gene *i *is defined by the geometric mean of all influence functions Ψ_*ij *_in a given pathway that involve gene *i *and satisfy Ψ_*ij *_> α:

where:

and:

The significance threshold, *α*, is used to control the contribution that gene *j *makes to *TIF*_*i*_. Note that shorter distances make an exponentially greater contribution to the mutual influence (and *TIF*) than do longer distances. The parameter α is used to control the sensitivity and selectivity of the *TIF*. After experimenting using the datasets studied in this report, the choice of α = 0.05 was found to represent a good apparent balance between sensitivity and selectivity. This parameter remains adjustable for future applications, however.

### Step 3

For all other genes from the 'Not *P*_*K*_' set, their *TIF *score is computed. Since topological information of genes from the 'Not *P*_*k*_' set is not available in pathway *k*, we use the central limit theorem to impute Ψ and *TIF *for each gene *i*. This procedure is theoretically sound, since the index of *TIF *score is actually an average of Ψ, which should follow the theory. (In practice, the imputations are done after all *TIFs *from all pathways are computed; that is, using the mean and variance from all pathways as the parameters for the background distribution of Ψ and *TIF*, not imputed just from one pathway. This sampling mitigates the bias of imputation when the size of the gene set is too small.) PWEA also measures the possibility of passing θ (i.e. having *f*_*ij *_≤ -ln α in the step function θ defined in Equation 4), and applies imputation only when a pass event happens. This is to maintain the distribution of all genes from being artificially altered after applying *TIF*, which is very likely to occur when it is applied only to genes in *P*_*K *_having topology. *TIF *scores for genes from the 'Not *P*_*K*_' set is important for fair ranking to avoid artificial bias toward genes in *P*_*K*_.

### Step 4

Calculate the statistical significance according to the WKS test. First, rank all genes by *r*_*j *_^1 + *TIF*^, where *r*_*j *_is the absolute value of the *t*-score (by *t*-test) of gene *j*. The *t*-test is performed on each gene to compare the expression levels between normal and disease samples. The cumulative distribution functions (CDFs) of *P*_*k *_and Not *P*_*k *_at position *i *in the rank can be written as:

and:

where  and *j *is the index of all genes belonging to *P*_*k*_.  is the number of genes belonging to Not *P*_*k *_and *k *is the index of all genes belonging to Not *P*_*k*_. The statistical significance for rejection of the null hypothesis is determined by comparing the maximum deviation (MD) of two cumulative distribution functions following *n *iterations of phenotype shuffling. Each randomly generated gene set for which the maximum deviation is higher than the original data will be counted, and after *n *iterations, the *P*-value is computed. In this work, *n *is set at 5,000 times.

### Step 5

After the *P*-values for all pathways are computed and the pathways have been ranked in ascending order, PWEA computes the FDR to correct for multiple testing [67]. Specifically, FDR = *P *× *m*/*k*, where *m *is the total number of pathways and *k *is the rank of the pathway under consideration.

### Step 6

A plain text file and a map file in KEGG markup language are produced. The map file represents the score of each gene in a color heatmap using the visANT software [68] (Figure S4 in Additional file 1).

The number of iterations, *n*, in step 4 must be sufficiently large, since PWEA simulates the background by random shuffling and the results may be biased if the sampling is insufficient. PWEA uses the absolute (that is, unsigned) metric when ranking genes. Use of an unsigned metric is important in many cases, especially KEGG pathways, which consist of multiple regulatory interactions. The signed metric used in the WKS test is designed for gene sets, such as chromosome segments that are expected to be up- or down-regulated under a given condition. Using an absolute metric can improve the clustering of high scoring genes and increase sensitivity. The parameter α, which appears in the *TIF*, can be adjusted by the user. Figure S6 in Additional file 1 demonstrates how the number of exclusively found pathways - which implies that the sensitivity changes - depends upon α. It can be seen that when α is large enough, PWEA reduces to GSEA, since *TIF *becomes zero and no weighting is applied.

PWEA has been implemented in a portable C++ package, and is freely available for download at [69]. The computing time is linear in the number of pathways, genes, and iterations of the permutation test. In this study, it took approximately 3 hours on one Sun Microsystems AMD 64 Opteron processor with 1 GB RAM for 201 pathways and 1,000 iterations for a dataset with about 10,000 genes. When a very large number of pathways and/or iterations must be carried out, a parallel version of PWEA, written with MPI [70], is available on the website above. The CPU time scales approximately linearly with the number of processors used. The output from PWEA can be visualized using visANT [71], which can give additional insight into the distribution of the high scoring genes.

## Abbreviations

AA: arachidonic acid; ALS: amyotrophic lateral sclerosis; COX: cyclooxygenase; EET: *cis*-epoxyeicosatrienoic acid; FDR: false discovery rate; GEO: Gene Expression Omnibus; GnRH: gonadotropin-releasing hormone; GO: Gene Ontology; GSEA: gene set enrichment analysis; IL: interleukin; KEGG: Kyoto Encyclopaedia of Genes and Genomes; K-S test: Kolmogorov-Smirnov statistic; MAPK: mitogen-activated protein kinase; PI3K: phosphoinositide 3-kinase; PWEA: pathway enrichment analysis; ROBO: roundabout receptor; TIF: topological influence factor; TNF: tumor necrosis factor; WKS: weighted Kolmogorov-Smirnov statistic.

## Authors' contributions

JHH designed and implemented the whole methodology and the computation framework. TWW provided constructive discussions, refinement of the formula and revised the manuscript. THY provided considerable statistical advice. ZH provided constructive discussions. ZW monitored the whole framework and revised the manuscript. CD directed the whole project, revised the manuscript, and is Principal Investigator on the NIH grant that funded the project. All the authors have read and agreed to the manuscript.

## Supplementary Material

Additional file 1 A Word document containing supplementary materials. Background knowledge of genetic buffering effect; comparison between different enrichment approaches; supplementary tables and figures. Click here for file

Additional file 2 A zip file containing the simulation output files of six test sets. Click here for file
