# Supplementary material for: Identification of functional modules that correlate with phenotypic difference: the influence of network topology
Source: Genome Biol. 2010 Feb 26;11(2):R23. doi: 10.1186/gb-2010-11-2-r23 (PMC2872883; doi:10.1186/gb-2010-11-2-r23)
Supplement: Additional file 1 — A Word document containing supplementary materials. Background knowledge of genetic buffering effect; comparison between different enrichment approaches; supplementary tables and figures. [file gb-2010-11-2-r23-S1.doc]

**Supplementary materials**

**Genetic buffering effect.** To further highlight the importance of using topological information in pathways, consider the two examples in Fig. S1. The first example in Fig. S1(a) illustrates a hypothetical pathway X, which involves six genes whose relations among one another are represented as arrows. The expressions of genes A and B are correlated, since gene A is the only activator of gene B. In contrast, genes A and C are distant from one another in the pathway and share no causal relationship; hence their expression should not be correlated. These differences between these two gene pairs should, therefore, be recognized when analyzing expression data associated with pathway X. In the second example, the impact that gene E plays on the pathway including genes A to D, as depicted in the left panel of Fig. S1(b), is called *genetic buffering* [61, 86]. To clarify, when a differentially expressed gene (in this case, gene A) fails to perturb any of its neighbors (or only very few; in this case, gene E only), it is possible that the impact this gene makes to this pathway is buffered by other genes in or outside of this pathway. Up-regulation of gene E can maintain the normal expression levels of genes B, C and D, when gene A is down-regulated. In contrast, the right panel in Fig. S1(b) depicts the same set of genes and regulatory relationships with the exception that genetic buffering is no longer present. In this case, repression of the expression of gene A has an effect upon genes B, C and D. Note that the two panels contain the same numbers of genes whose expression levels are up-regulated, down-regulated, and unchanged. It is useful to be able to distinguish between these two cases within an expression data analysis protocol, which is only possible when the topological information of the pathway is taken into account.

**Comparison with other enrichment approaches.** For further understanding of the prediction power of PWEA, we listed the predicted results of Pathway Express and the Hypergeometric test in Table S2. For Pathway Express, the inputs consist of the differentially expressed gene list and the fold-change in expression that is associated with each gene. The fold-change is expressed as log_2_(ratio) (ratio = mean of disease expression level/mean of normal expression level), and only genes with log_2_(ratio) > 1 will appear in the differential expression list. For the Hypergeometric test, the inputs are the numbers of differentially expressed genes within each gene sets and the background. Differentially expressed genes are those with t-test *p-value* < 0.05.

**Figures**


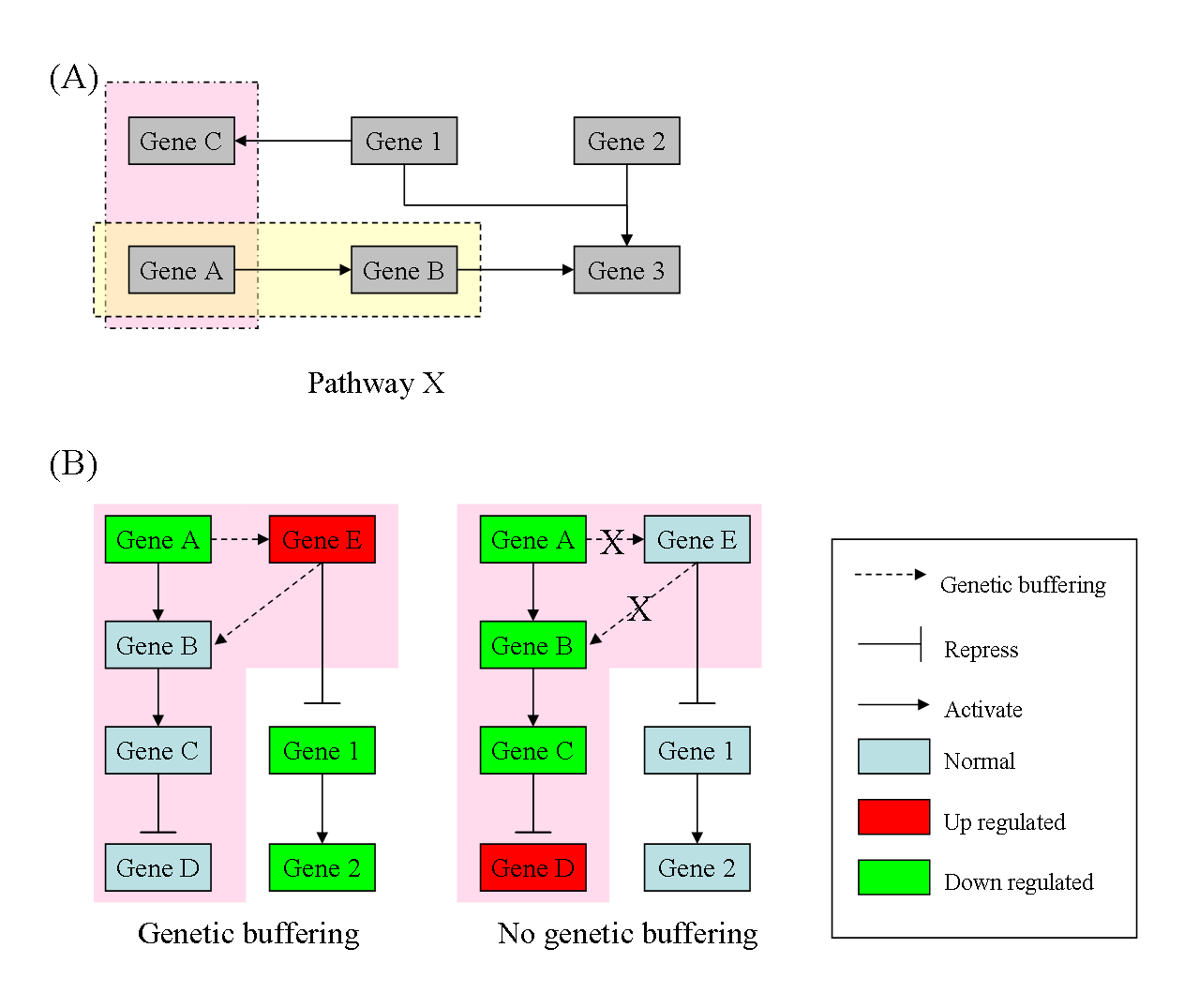


Figure S1. (A) In pathway X, because gene A directly regulates B, one expects that their expression profiles should be more correlated than are those of genes A and C. (b) Two different pathways containing the same set of genes with different topologies. As noted in the legend, the color of each gene indicates its differential expression level. Assume that gene A is being knocked down and that gene D is the reporter that indicates the functionality of this pathway. In the case where no genetic buffering is present (pink-background region of the right panel), every gene downstream of gene A in the pathway is affected by the expression change of gene A. In contrast, when genetic buffering is present (pink-background region of the left panel), gene E acts as a buffer to maintain the normal expression levels of genes B, C and D. These two cases would be indistinguishable to GSEA or similar methods, which rank pathways solely based on the differential expression levels of the component genes, because there are three up-regulated genes and one down-regulated gene in both panels.


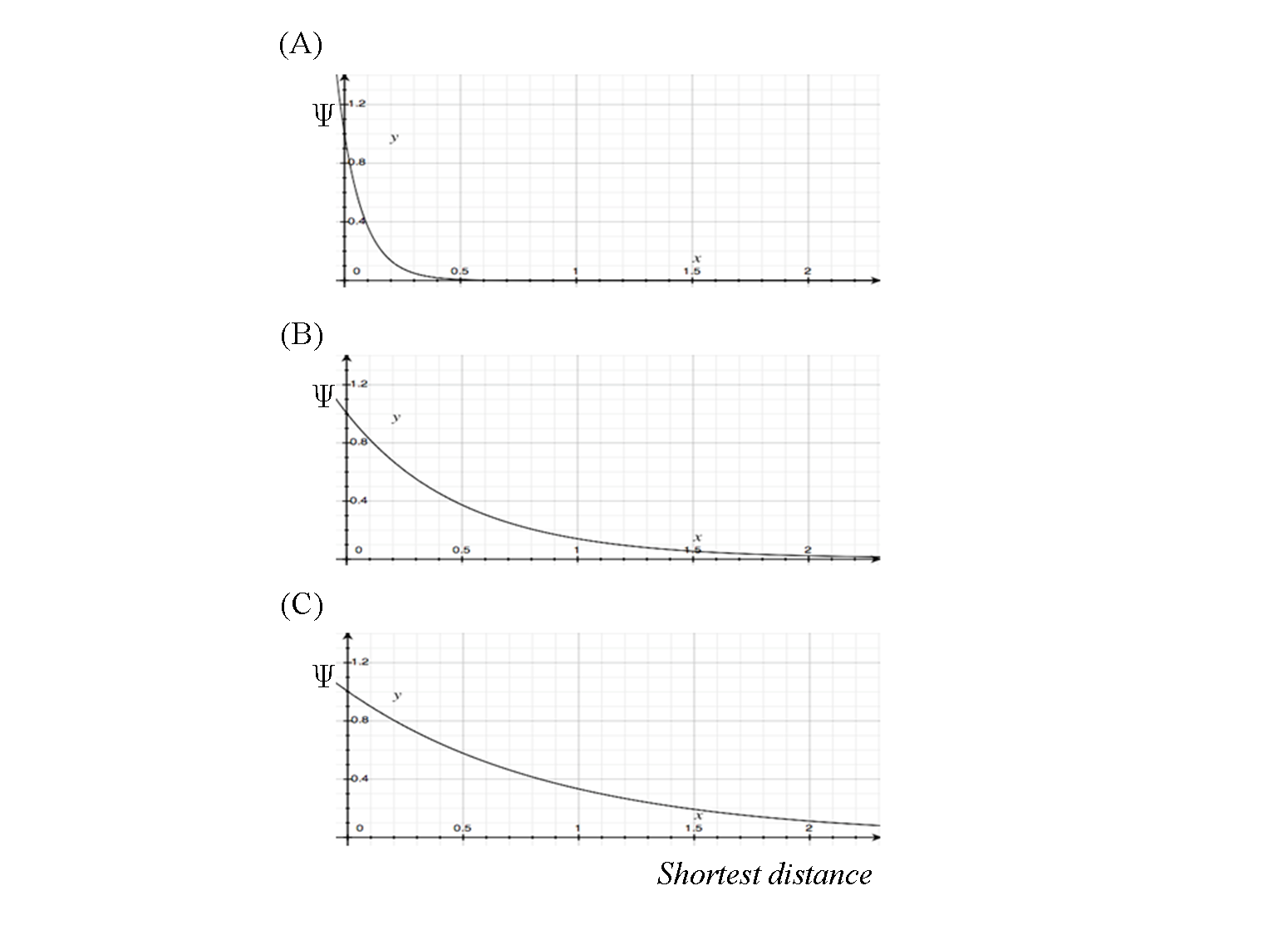


Figure S2. The behavior of *mutual influence* (Ψ_ij_). X axis is the distance between gene i and gene j. (A) when c= 0.1, (B) when c=0.5, and (C) when c=0.9.


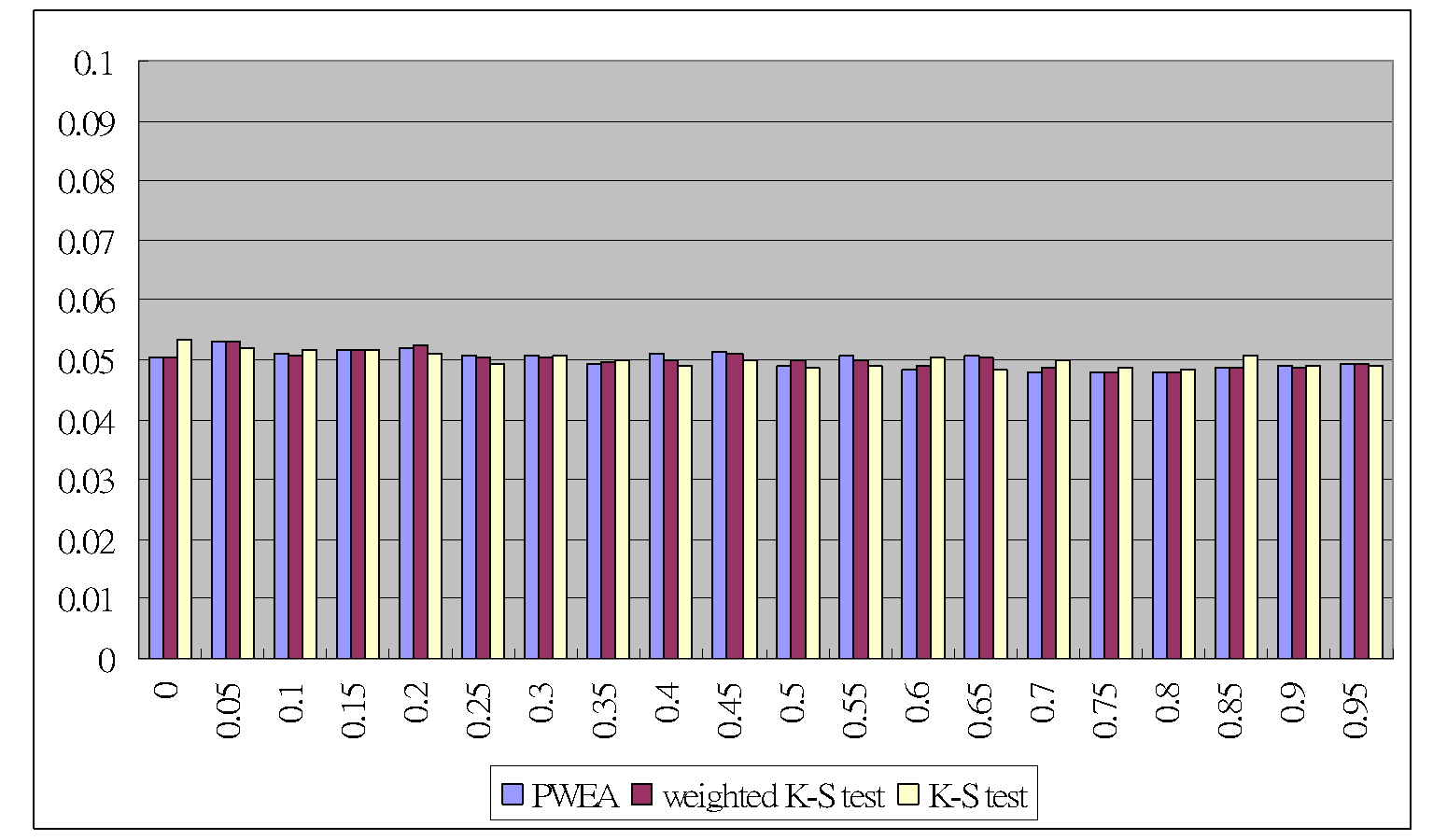


Figure S3. The distribution of *p-value* from 500 random shuffled input with different methods.


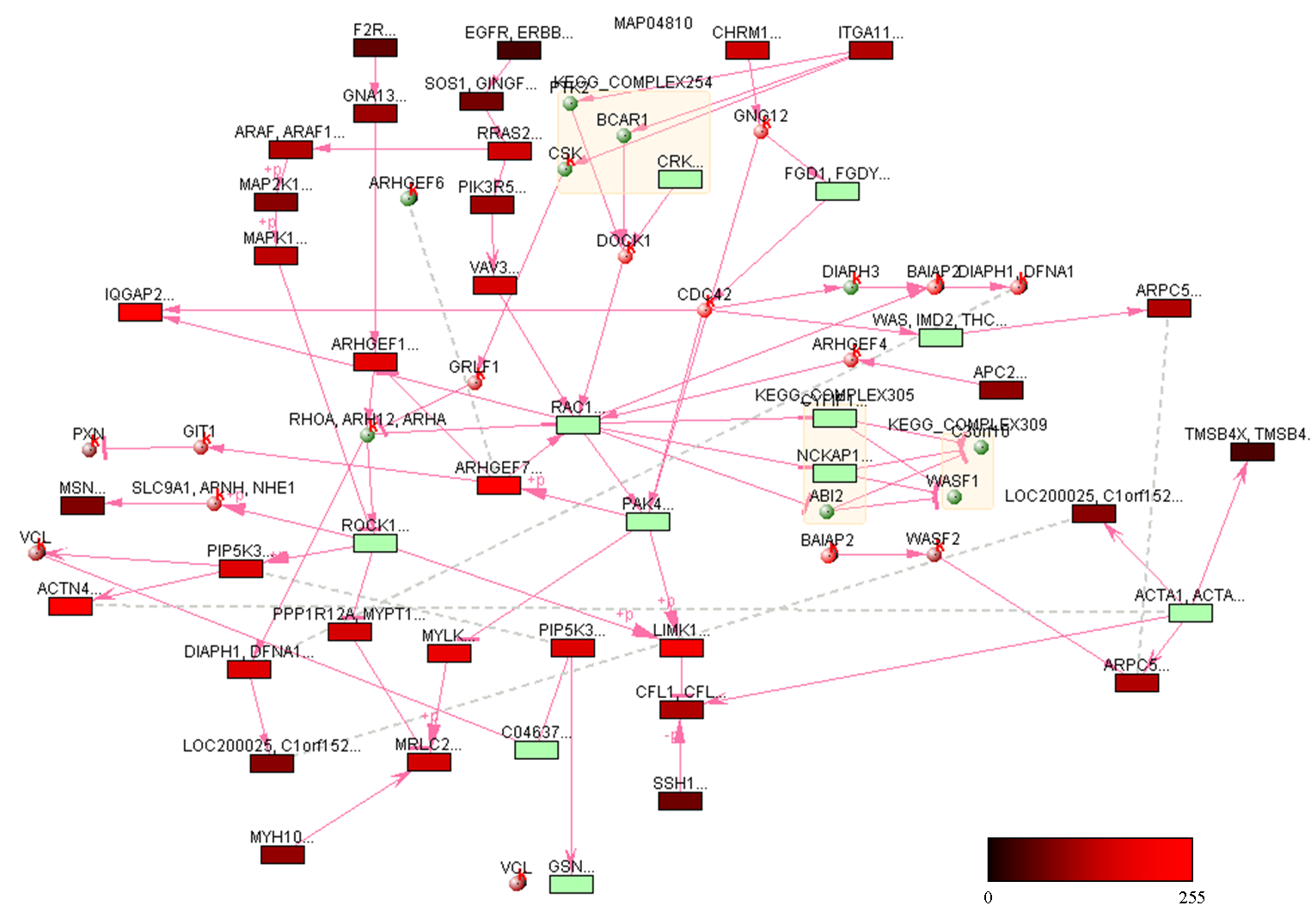


Figure S4. Visualization of the density score, generated by VisANT, for each gene in the *regulation of actin cytoskeleton* pathway. Genes are represented as spheres and the interactions among them as lines. When multiple genes (typically members of the same family) participate in the same interactions, they are collapsed into a rectangle (see http://visant.bu.edu/ for more details). Genes with zero scores are in green.

Figure S5. The KS statistics showing no bias distribution of p values with different gene set size in our 500 simulations. X axis is the size of pathways and y is the KS statistics, none of them shows significant deviation.

Figure S6. The numbers of exclusive pathways found by PWEA comparing to GSEA when using different α among 4 datasets. X axis is the value of α used in PWEA, and Y axis is the number of exclusive pathways found by PWEA.

**Tables**

Table S1. All pathways found by PWEA in all datasets with FDR corrected *p-values* < 0.01. Pathways in bold were reported by both methods (PWEA and conventional WKS test).

| **Colon cancer dataset** | **Small cell lung cancer dataset** | **Ovarian endometriosis** | **Rheumatoid arthritis** | **Parkinson’s disease** |
| --- | --- | --- | --- | --- |
| **Valine, leucine and isoleucine degradation** | **Bile acid biosynthesis** | **ErbB signaling pathway** | Antigen processing and presentation | **VEGF signaling pathway** |
| **Butanoate metabolism** | **Focal adhesion** | **Calcium signaling pathway** | -- | -- |
| **MAPK signaling pathway** | **Tight junction** | **Wnt signaling pathway** |  |  |
| **Calcium signaling pathway** | **PPAR signaling pathway** | **Adherens junction** |  |  |
| **ErbB signaling pathway** | **Gap junction** | **Pathways in cancer** |  |  |
| **Gap junction** | **Tyrosine metabolism** | **Endometrial cancer** |  |  |
| **beta-Alanine metabolism** | **Cytokine-cytokine receptor interaction** | **Glioma** |  |  |
| **Limonene and pinene degradation** | **Leukocyte transendothelial migration** | **Melanoma** |  |  |
| **Lysine degradation** | **Pathways in cancer** | **Chronic myeloid leukemia** |  |  |
| **Regulation of actin cytoskeleton** | **Hematopoietic cell lineage** | **Ether lipid metabolism** |  |  |
| **GnRH signaling pathway** | **ECM-receptor interaction** | **MAPK signaling pathway** |  |  |
| **Pathways in cancer** | **Cell adhesion molecules (CAMs)** | **mTOR signaling pathway** |  |  |
| **Fatty acid metabolism** | **Small cell lung cancer** | **Apoptosis** |  |  |
| **PPAR signaling pathway** | **Retinol metabolism** | **Tight junction** |  |  |
| **Renal cell carcinoma** | **Systemic lupus erythematosus** | **Insulin signaling pathway** |  |  |
| **Tryptophan metabolism** | **Regulation of actin cytoskeleton** | **Colorectal cancer** |  |  |
| **Propanoate metabolism** | **Arachidonic acid metabolism** | **Pancreatic cancer** |  |  |
| **3-Chloroacrylic acid degradation** | **Melanogenesis** | **Prostate cancer** |  |  |
| **Focal adhesion** | **Adherens junction** | **Non-small cell lung cancer** |  |  |
| **Adherens junction** | GnRH signaling pathway | **Urea cycle and metabolism of amino groups** |  |  |
| **Epithelial cell signaling in Helicobacter pylori infection** | Complement and coagulation cascades | **Ubiquitin mediated proteolysis** |  |  |
| **Ascorbate and aldarate metabolism** | Histidine metabolism | **Axon guidance** |  |  |
| **Wnt signaling pathway** | Fatty acid metabolism | **Focal adhesion** |  |  |
| **Fatty acid elongation in mitochondria** | Phenylalanine metabolism | **Cell cycle** |  |  |
| Bile acid biosynthesis | Jak-STAT signaling pathway | **Regulation of actin cytoskeleton** |  |  |
| Long-term depression | Tryptophan metabolism | **GnRH signaling pathway** |  |  |
| Arachidonic acid metabolism | Basal cell carcinoma | **Acute myeloid leukemia** |  |  |
| Colorectal cancer | MAPK signaling pathway | **Thyroid cancer** |  |  |
| Urea cycle and metabolism of amino groups | Fc epsilon RI signaling pathway | **Cell adhesion molecules (CAMs)** |  |  |
| Axon guidance | Drug metabolism - other enzymes | **Gap junction** |  |  |
| Nicotinate and nicotinamide metabolism | Apoptosis | **Lysine degradation** |  |  |
| Drug metabolism - cytochrome P450 | ABC transporters | **Glutathione metabolism** |  |  |
| Amyotrophic lateral sclerosis (ALS) | Drug metabolism - cytochrome P450 | **Long-term potentiation** |  |  |
| Pyruvate metabolism | -- | Urea cycle and metabolism of amino groups |  |  |
| -- |  | Type II diabetes mellitus |  |  |
|  |  | Sphingolipid metabolism |  |  |
|  |  | Small cell lung cancer |  |  |
|  |  | Long-term depression |  |  |
|  |  | Glycolysis / Gluconeogenesis |  |  |
|  |  | Nitrogen metabolism |  |  |
|  |  | Complement and coagulation cascades |  |  |
|  |  | Purine metabolism |  |  |

Table S2. Pathways found by Pathway Express, and Hypergeometric test in two cancer datasets. Pathways are reported to be significant for FDR<0.01. Pathways identified by two or more methods are in bold.

| **Dataset** | **Pathways with FDR < 0.01** | |
| --- | --- | --- |
|  | Pathway-Express* | Hypergeometric test |
| **Colorectal cancer** | **Cell adhesion molecules (CAMs)** | **Fatty acid metabolism** |
|  | Phosphatidylinositol signaling system | **Valine, leucine and isoleucine degradation** |
|  | Antigen processing and presentation | **Propanoate metabolism** |
|  | Adherens junction | **beta-Alanine metabolism** |
|  | Circadian rhythm | **Ascorbate and aldarate metabolism** |
|  | -- | **Butanoate metabolism** |
|  | -- | Glycolysis / Gluconeogenesis |
|  | -- | Oxidative phosphorylation |
|  |  | Parkinson's disease |
| **Small cell lung cancer** | **Cell adhesion molecules (CAMs)** | **Systemic lupus erythematosus** |
|  | Phosphatidylinositol signaling system | Asthma |
|  | Antigen processing and presentation | -- |

*Pathway Express used the older version of KEGG pathways, so the results are not totally comparable.

Table S3. The uniquely pathways found by PWEA and GSEA in four non-cancer datasets. All GSEA’s discoveries are covered by PWEA.

| **Dataset** | **Pathways with FDR < 0.01** | |
| --- | --- | --- |
|  | PWEA | GSEA* |
| **Ovary**  **endometriosis** | Urea cycle and metabolism of amino groups | -- (33) |
|  | Type II diabetes mellitus |  |
|  | Sphingolipid metabolism |  |
|  | Small cell lung cancer |  |
|  | Long-term depression |  |
|  | Glycolysis / Gluconeogenesis |  |
|  | Nitrogen metabolism |  |
|  | Complement and coagulation cascades |  |
|  | Purine metabolism |  |
| **Rheumatoid arthritis** | Antigen processing and presentation | --(0) |
| **Parkinson’s Disease** | -- | --(1) |
| **Sex** | -- | --(0) |

*The number in the parenthesis indicates the number of significant pathways that GSEA found.

Table S4. The comparison of the numbers of found pathways using three simple gene set level statistics [13] (mean, medium and Wilcoxon rank sum test) with and without *TIF* weighting (i.e. applying PWEA) in all datasets. In general, PWEA’s weighting scheme improves the sensitivity among all. Numbers in red if the weighting shows improvement.

| **Dataset** | **Pathways with FDR < 0.01** | | | | | |
| --- | --- | --- | --- | --- | --- | --- |
|  | **PWEA**  **(Mean)** | Mean | **PWEA**  **(Medium)** | Medium | **PWEA**  **(Wilcoxon)** | Wilcoxon |
| **Colon Cancer** | **140** | 136 | **123** | 115 | 74 | 75 |
| **Small cell lung cancer** | **126** | 119 | **103** | 102 | **80** | 73 |
| **Ovary**  **endometriosis** | **129** | 119 | 122 | 125 | **87** | 81 |
| **Rheumatoid arthritis** | **54** | 50 | **30** | 28 | **36** | 35 |
| **Parkinson’s Disease** | **47** | 37 | **39** | 38 | 25 | 25 |
| **Sex** | 0 | 0 | 0 | 0 | 0 | 0 |
